# The role of TACE in the era of immune-targeted therapy for hepatocellular carcinoma: a meta-analysis based on PSM

**DOI:** 10.3389/fimmu.2025.1573834

**Published:** 2025-04-02

**Authors:** Jiahao Li, Lei Xian, Xinsen Wang, Yingnan Liu, Jiarui Li

**Affiliations:** ^1^ Department of Interventional Therapy, The First Hospital of Jilin University, Changchun, Jilin, China; ^2^ Department of Radiology, The First Hospital of Jilin University, Changchun, Jilin, China

**Keywords:** hepatocellular carcinoma, transarterial chemoembolisation, immune-targeted therapy, meta-analysis, combination therapy

## Abstract

**Background:**

Hepatocellular carcinoma (HCC) is a major global health challenge, with over 50% of patients ineligible for curative treatments at diagnosis. The combination of molecular targeted therapies and immunotherapy has shown promise in improving outcomes for advanced HCC.

**Objective:**

This meta-analysis aims to assess the efficacy of combining transarterial chemoembolisation (TACE) with immune-targeted therapies in patients with unresectable HCC.

**Methods:**

A systematic review and meta-analysis conforming to PRISMA guidelines were conducted by searching PubMed, Embase, Web of Science, and the Cochrane Library for studies published up to January 5, 2025. Due to the limited clinical evidence, our study exclusively included retrospective studies based on propensity score matching (PSM) analysis that compared the efficacy of TACE in combination with immune-targeted therapy to immune-targeted therapy alone. Key outcomes assessed included objective response rate (ORR), disease control rate (DCR), one-year overall survival (1-OS), one-year progression-free survival (1-PFS), median overall survival (mOS), and median progression-free survival (mPFS).

**Results:**

A total of 9 PSM studies involving 2119 patients were included. The meta-analysis revealed that TACE significantly improved ORR, DCR, 1-OS, and 1-PFS, in addition to extending mOS and mPFS.

**Conclusion:**

The findings suggest that the inclusion of TACE in treatment regimens for unresectable HCC notably enhances tumour control and patient survival. This study provides moderate to high-quality evidence supporting the integration of TACE in advanced HCC management, particularly for those patients not meeting standard TACE criteria.

**Systematic review registration:**

https://www.crd.york.ac.uk/PROSPERO/, identifier CRD 42025631817.

## 1 Introduction

Primary liver cancer is a significant global health issue, ranking as one of the top three causes of cancer-related mortality in 46 countries and one of the top five in 90 countries worldwide in 2020 (1). Upon initial diagnosis, more than 50% of patients are deemed ineligible for curative treatment and are instead candidates for systemic or locoregional therapies (2, 3).

In recent years, molecular targeted therapies and immunotherapy have significantly improved the management of patients with advanced hepatocellular carcinoma (HCC) (4–6). Several large Phase III clinical trials have demonstrated the advantages of combination treatment with immune checkpoint inhibitors and tyrosine kinase inhibitors/anti-VEGF antibodies. Consequently, the combination of molecular targeted therapies and immunotherapy has been recommended as a first-line treatment option for advanced HCC.

Transarterial chemoembolisation (TACE) is a local regional therapy that combines embolisation and chemotherapy, exerting its anti-cancer effects by selectively occluding the tumour blood supply arteries and delivering high concentrations of chemotherapeutic agents. According to the Barcelona Clinic Liver Cancer (BCLC) staging system, most guidelines and consensus recommend TACE as the standard first-line treatment for intermediate hepatocellular carcinoma (HCC) (BCLC B) (7–9). Immune microenvironment remodelling and tumour angiogenesis are associated with tumour progression following TACE (10), providing a theoretical basis for the combined application of TACE with molecular targeted therapies and immunotherapy. Several studies have supported the advantages of combining TACE with immune-targeted therapies compared to TACE alone (11, 12). Furthermore, some meta-analyses based on retrospective studies have highlighted the prospects of transarterial therapies in conjunction with immune-targeted treatments (13, 14), although broad inclusion criteria have limited the quality of the evidence. For patients with unresectable HCC, particularly non-TACE candidates, such as those meeting the up-to-seven criteria for intermediate HCC and patients with advanced HCC, whether the combination of TACE with immune-targeted treatment confers survival benefits still requires strong substantiation. Therefore, we conducted a meta-analysis aimed at investigating the efficacy of immune-targeted treatments with or without the addition of TACE in the management of HCC and providing higher-quality evidence to support this combined approach.

## 2 Methods

### 2.1 Protocol and registration

This systematic review and meta-analysis complied with the Preferred Reporting Items for Systematic Review and Meta-Analyses (PRISMA) statement (**Supplementary Table S1**). The protocol for this meta-analysis was registered with PROSPERO (ID: CRD 42025631817).

### 2.2 Data sources and search strategy

We conducted an extensive search of multiple electronic databases, including PubMed, Embase, Web of Science, and the Cochrane Library, to identify relevant studies published from the establishment of the database up to January 5, 2025. The search strategy is detailed in **Supplementary Table S2**. For the identified studies and relevant reviews, we manually searched their reference lists to determine potential related research. No language restrictions were applied to the search.

### 2.3 Inclusion and exclusion criteria

Inclusion criteria for published studies that adhere to the following PICOS principles:

#### 2.3.1 Population

Participants diagnosed with hepatocellular carcinoma (HCC).

#### 2.3.2 Intervention

Combination therapy of immune-targeted therapy with transarterial chemoembolization (TACE).

#### 2.3.3 Comparison

Combined therapies involving targeted therapies (TKIs or VEGF antibodies) with immune checkpoint inhibitors.

#### 2.3.4 Outcomes

Trials reporting one or more of the following clinical outcomes:

- Objective Response Rate (ORR), defined per mRECIST as the proportion of patients with complete or partial response after treatment relative to the total number of patients treated.
- Disease Control Rate (DCR), defined per mRECIST as the proportion of patients with complete response, partial response, and stable disease after treatment relative to the total number of patients treated.
- 1-Year Overall Survival (1-OS), the proportion of patients who are alive one year after treatment.
- 1-Year Progression-Free Survival (1-PFS), the proportion of patients without tumour progression or death one year after treatment.
- Median Progression-Free Survival (mPFS), defined as the time from the initiation of treatment until half of the patients experience disease progression or death.
- Median Overall Survival (mOS), defined as the time from the commencement of treatment until the death of half of the patients.

#### 2.3.5 Study type

Randomized controlled trials, prospective cohort studies, or propensity score matched (PSM) analyses.

Additionally, we excluded any trial protocols, reviews, case series, abstracts, duplicate data, and non-PSM retrospective studies.

### 2.4 Data extraction and quality assessment

The research team prepared a standardized Excel spreadsheet in advance for data extraction. The extracted data included the first author, year of publication, region, and sample size of the study; basic characteristics of the patient cohort, including age, sex ratio, HBV infection status, Child-Pugh classification, tumour size, number of tumours, and presence of metastasis; as well as outcome data, including ORR, DCR, 1-OS, 1-PFS, mOS, and mPFS. Data from survival curves were extracted using Engauge Digitizer software if detailed reports were not available in the text.

The ROBINS-I tool was used to assess the risk of bias in non-randomised controlled trials (15). Compared to the Newcastle-Ottawa Scale, the ROBINS-I tool is more detailed and rigorous, and it aligns well with the GRADE system. evaluating seven domains before, during, and after the intervention, which include confounding bias, selection bias of participants, classification bias of interventions, bias due to deviations from intended interventions, missing data bias, measurement bias of outcomes, and selective reporting bias (15). The quality assessment process was conducted independently by two members of the research team, with any disagreements resolved through discussion among all team members. Finally, the GRADE approach was employed to assess the quality of evidence for the combined results (https://www.gradepro.org).

### 2.5 Statistical analysis

The primary outcome measures were ORR, DCR, 1-OS, and 1-PFS, while the secondary outcomes included mOS and mPFS. Binary outcome measures were calculated using odds ratios (OR) and their corresponding 95% confidence intervals. Survival outcome measures were determined using hazard ratios (HR) and their 95% confidence intervals. A random-effects model was employed for the meta-analysis. During the combined analysis, heterogeneity among the included studies was assessed using I² statistics. For outcomes with substantial heterogeneity, sensitivity analyses, subgroup analyses, and meta-regression were conducted to explore the potential sources of heterogeneity. Additionally, publication bias was assessed using funnel plots and Egger’s regression test; when significant publication bias was detected, trim-and-fill methods were applied to adjust the pooled results. All statistical analyses were conducted using the ‘meta’ package in R software.

Furthermore, for the analysis of the primary outcome measures, we conducted a sequential trial analysis (TSA) to control for type I and type II errors in the pooled results. We defined 5% type I error rate (two-sided) and 80% statistical power to calculate the traditional boundaries. The relative risk reduction (RRR) was determined based on published studies (16). TSA was performed using the TSA 0.9.5.10 Beta software.

## 3 Results

### 3.1 Study selection and characteristics

**Figure 1:**
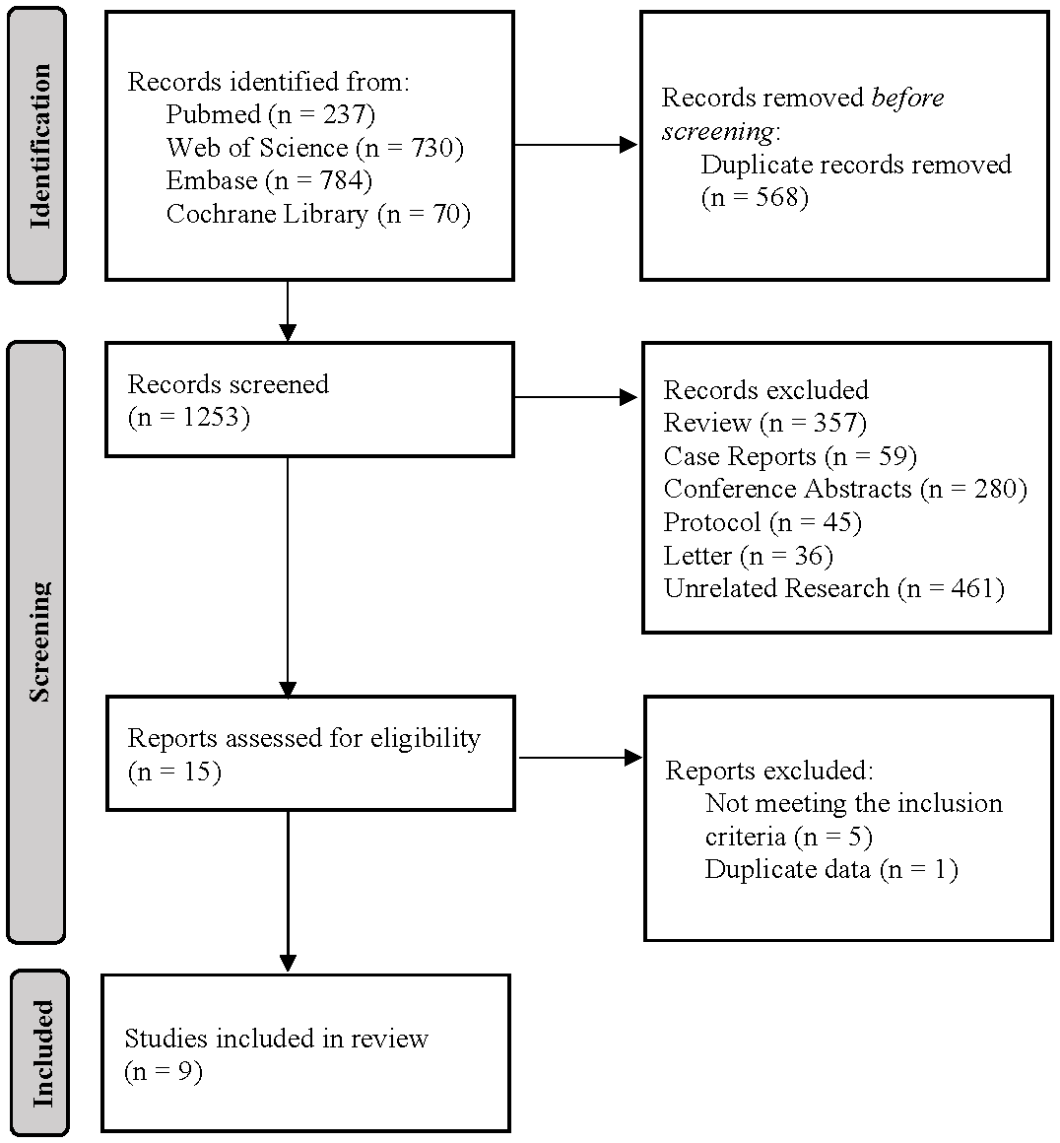
The process of literature review and selection.

A preliminary search of electronic databases yielded a total of 1821 literature records. After removing duplicates and reviewing titles and abstracts, 15 studies were considered for inclusion based on the eligibility criteria. Following the exclusion of non-PSM retrospective studies and those with duplicated data, 9 non-randomised controlled trials were included (3, 17–24). Refer to **Figure 1** for details.

All nine non-randomised controlled trials were conducted in China and collectively included 2119 patients diagnosed with HCC. Among these studies, seven employed PSM analysis, while two utilised stabilised inverse probability of treatment weighting (sIPTW) to balance intergroup differences. Post-matching, there were no significant intergroup discrepancies, indicating strong comparability between groups. Of the included studies, five were multicentre investigations. Four studies specifically focused on patients with advanced HCC, whose lesions could not be completely treated with TACE. Consequently, incomplete embolisation became more prevalent in these investigations. It is noteworthy that three studies excluded or did not include patients with main portal vein invasion in their cohorts, whereas the remaining six studies did not report the proportion of patients with main portal vein invasion in their populations. Furthermore, within these studies, eight reported that TACE was administered on an “on-demand” basis, determined through comprehensive evaluations during follow-up assessments based on liver function and disease progression, while one study conducted TACE according to a predetermined schedule, with patients receiving repeated TACE treatments at one-month intervals. Exclusion criteria across all studies included patients lost to follow-up or with missing data; however, only two studies reported the number of excluded patients, which represents a significant source of bias risk. The basic characteristics of all included studies are summarised in **Supplementary Table S3**. The methodological quality of all studies was assessed using the ROBINS-I tool, which indicated a low to moderate risk of bias, as detailed in **Supplementary Table S4**.

### 3.2 ORR

**Figure 2:**
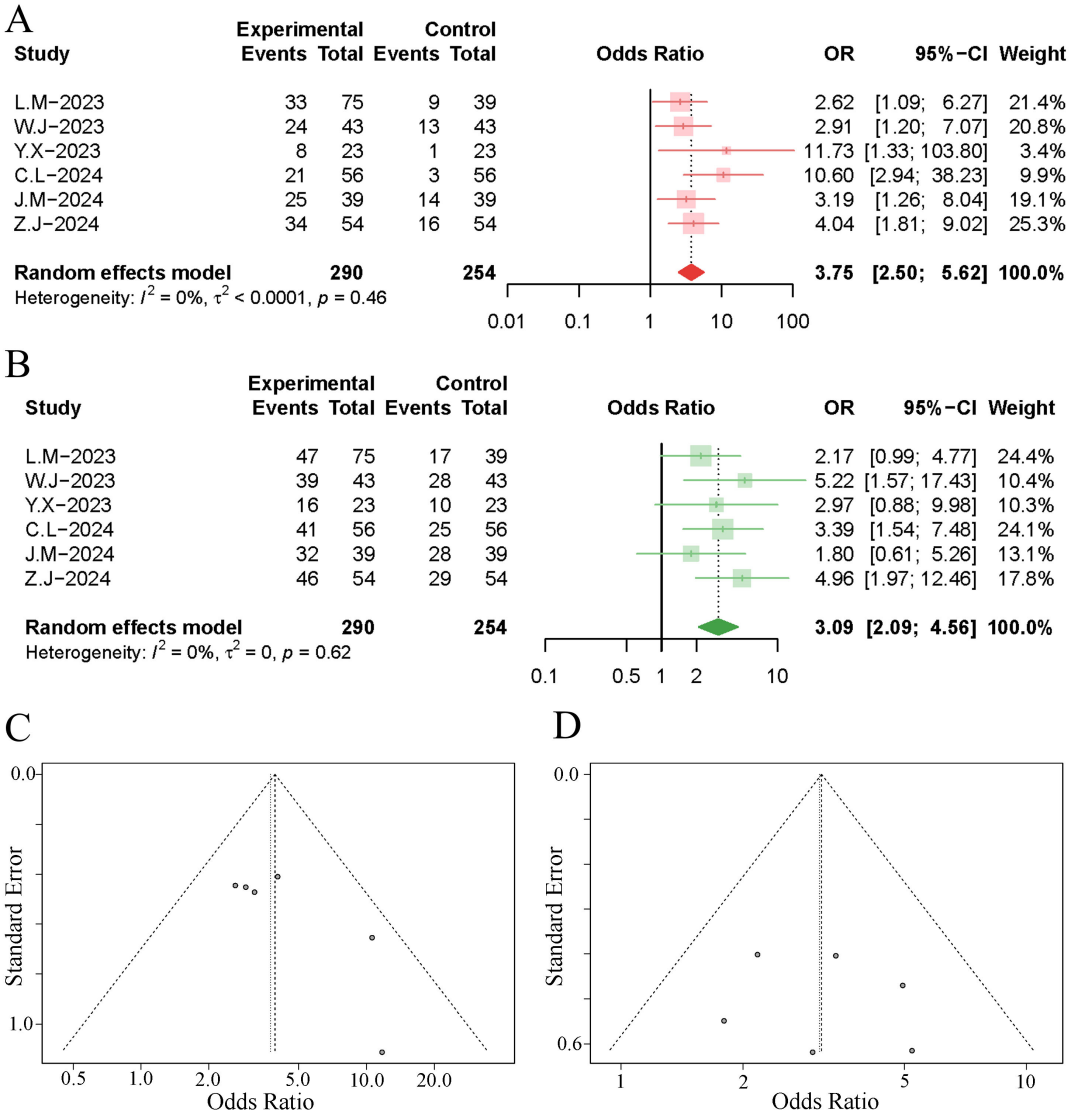
Forest plot and funnel plot of the meta-analysis results. **(A)** forest plot of ORR; **(B)** forest plot of DCR; **(C)** funnel plot of ORR; **(D)** funnel plot of DCR.

6 studies reported the objective response rates at 1 to 3 months post-treatment. The meta-analysis indicated that TACE significantly increased the objective response rate of tumours in HCC patients undergoing immuno-targeted therapy, with no significant heterogeneity among the studies (OR 3.75, 95% [2.50, 5.62]; I²=0%, p=0.46). Neither the funnel plot nor Egger’s test revealed significant publication bias (p=0.081). Further details are presented in **Figures 2A, C**.

### 3.3 DCR

6 studies reported the disease control rates at 1 to 3 months post-treatment. The results of the meta-analysis demonstrated that the implementation of TACE significantly increased the disease control rate of tumours in HCC patients undergoing immuno-targeted therapy, with no significant heterogeneity observed among the studies (OR 3.09, 95% [2.09, 4.56]; I²=0%, p=0.62). Neither the funnel plot nor Egger’s test revealed significant publication bias (p=0.741). Further details are presented in **Figures 2B, D**.

### 3.4 1-OS

**Figure 3:**
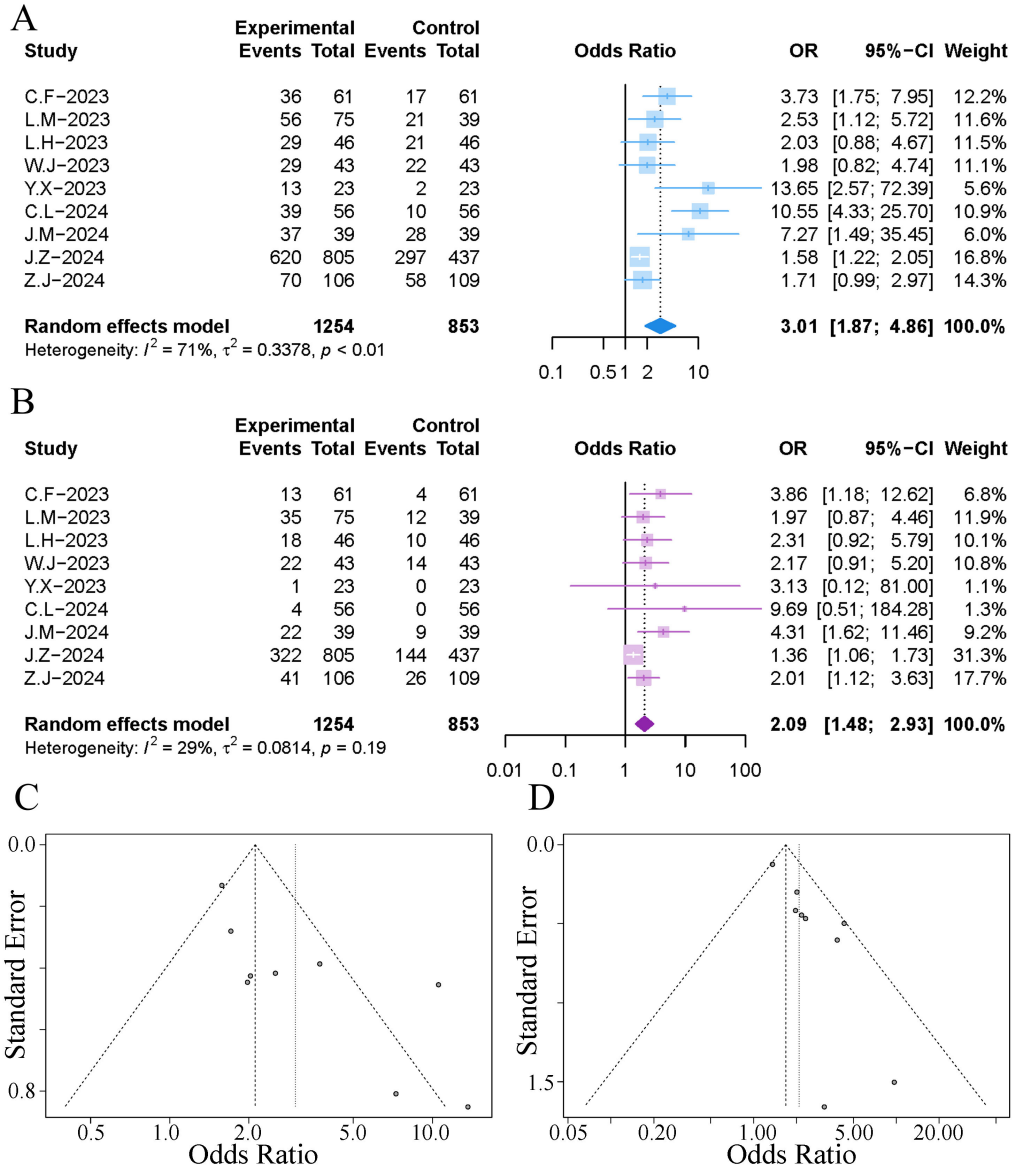
Forest plot and funnel plot of the meta-analysis results. **(A)** forest plot of 1-year OS; **(B)** forest plot of 1-year PFS; **(C)** funnel plot of 1-year OS; **(D)** funnel plot of 1-year PFS.

All studies reported the survival rates at one year post-treatment. The results of the meta-analysis indicated that the addition of TACE during immuno-targeted therapy significantly increased the one-year survival rate of patients (OR 3.01, 95% CI [1.87, 4.86]). However, the combined results exhibited significant heterogeneity (I²=71%, p<0.01), as illustrated in **Figure 3**. Sensitivity analysis conducted to explore the source of heterogeneity revealed that the exclusion of the study by Cao et al. significantly reduced the heterogeneity, as shown in **Supplementary Figure 1A**. Subsequent analysis of the funnel plot and Egger’s test indicated a notable publication bias (p=0.012), as depicted in **Figure 3**. We employed a trim-and-fill method to correct for publication bias while excluding the study by Cao et al., in order to minimise the impact of small-study effects on the combined results (25). The trim-and-fill method identified an additional four publications (**Supplementary Figure 2A**), and the adjusted results diminished the effect of immuno-targeted therapy combined with TACE, yet still showed a statistically significant difference (OR 1.71, 95% CI [1.09, 2.67]).

### 3.5 1-PFS

All studies reported the recurrence-free survival rates at one year post-treatment. The results of the meta-analysis indicated that the addition of TACE during immuno-targeted therapy significantly increased the one-year recurrence-free survival rate of patients, with no significant heterogeneity observed (OR 2.09, 95% CI [1.48, 2.93]; I²=29%, p=0.19), as illustrated in **Figure 3**. Analysis of the funnel plot and Egger’s test revealed significant publication bias (p=0.002), as depicted in **Figure 3**. The trim-and-fill method identified an additional five publications (**Supplementary Figure 2B**), and the adjusted combined results still exhibited a statistically significant difference (OR 1.62, 95% CI [1.16, 2.28]).

### 3.6 mOS

**Figure 4:**
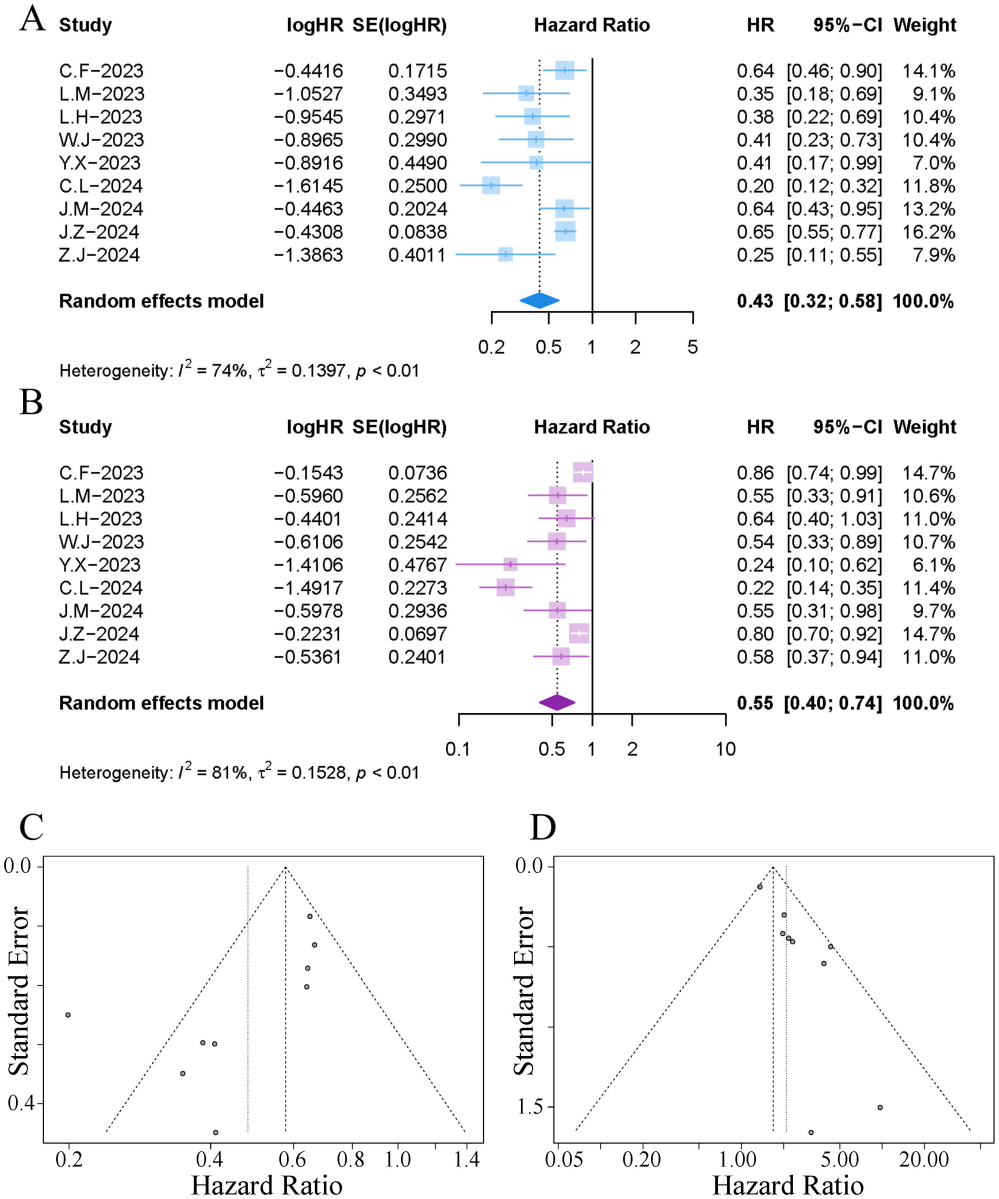
Forest plot and funnel plot of the meta-analysis results. **(A)** forest plot of OS; **(B)** forest plot of PFS; **(C)** funnel plot of OS; **(D)** funnel plot of PFS.

All studies reported data on mOS. The results of the meta-analysis indicated that the implementation of TACE in patients with HCC receiving immuno-targeted therapy significantly extended the median survival time; however, significant heterogeneity was observed among the studies (HR 0.43, 95% CI [0.32, 0.58]; I²=74%, p<0.01), as illustrated in **Figure 4**. Sensitivity analysis revealed that the exclusion of the study by Cao et al. significantly reduced the heterogeneity, as shown in **Supplementary Figure S1B**. The funnel plot indicated notable publication bias, and Egger’s test yielded a p-value of 0.039. The trim-and-fill method identified an additional two publications (**Supplementary Figure S2C**), and the adjusted combined results continued to demonstrate a statistically significant difference (HR 0.63, 95% CI [0.57, 0.71]).

### 3.7 mPFS

All studies reported data on mPFS. The results of the meta-analysis indicated that the implementation of TACE in patients with HCC receiving immuno-targeted therapy significantly extended the mPFS; however, significant heterogeneity was observed among the studies (HR 0.55, 95% CI [0.40, 0.74]; I²=81%, p<0.01), as illustrated in **Figure 4**. Sensitivity analysis revealed that the exclusion of the study by Cao et al. significantly reduced the heterogeneity. The funnel plot indicated notable publication bias, and Egger’s test yielded a p-value of 0.014. The trim-and-fill method identified an additional four publications (**Supplementary Figure S2D**), and the adjusted combined results continued to demonstrate a statistically significant difference (HR 0.78, 95% CI [0.62, 0.96]).

### 3.8 Subgroup analysis and regression analysis

4 studies included only patients with advanced hepatocellular carcinoma (BCLC stage C), characterised by extensive vascular invasion or extrahepatic metastasis. Among these studies, the proportion of patients with vascular invasion ranged from 53.28% to 100%, while those with extrahepatic metastasis comprised between 26.51% and 58.6%. The results of the subgroup analysis revealed that in these patient cohorts, the combination of immunotherapy with targeted therapy and TACE yielded better outcomes than immunotherapy with targeted therapy alone for 1-OS (OR 1.87, 95% CI [1.35, 2.59]; I²=33%, p=0.21), 1-PFS (OR 1.77, 95% CI [1.20, 2.60]; I²=36%, p=0.19), mOS (HR 0.64, 95% CI [0.56, 0.72]; I²=0%, p=0.50), and mPFS (HR 0.80, 95% CI [0.73, 0.88]; I²=39%, p=0.18), although the benefits were reduced compared to the combined results that included patients with intermediate to advanced HCC (**Supplementary Figures S3**, **4**). It is important to note that these studies did not include patients with invasion of the main portal vein, a specific subgroup often associated with very poor prognosis, for whom the implementation of TACE is limited.

Furthermore, subgroup analyses based on the proportions of vascular invasion and extrahepatic metastasis within the patient cohorts consistently demonstrated that the combination of immunotherapy and targeted therapy with TACE significantly enhances patients’ 1-OS, 1-PFS, mOS, and mPFS when compared to immunotherapy and targeted therapy alone (**Supplementary Figures 5–8**).

The meta-regression analysis for 1-OS indicated that the gender ratio and HBV infection rate within the patient cohorts might be sources of heterogeneity, explaining 60.89% and 63.61% of the heterogeneity, respectively. The meta-regression analysis of the combined results for mOS and mPFS showed that study sample size, gender ratio, HBV infection rate, Child-Pugh classification, vascular invasion, and extrahepatic metastasis did not have a significant effect on the combined results, further confirming the robustness of the meta-analysis model (**Table 1**).

**Table 1:** Results of the meta-regression analysis.

	1-OS	OS	PFS
**Sample Size**	-0.0007 (-0.0018, 0.0003)	0.0003 (-0.0003, 0.0010)	0.0004 (-0.0003, 0.0012)
**Sex**	-0.1791 (-0.3063, -0.0520)*	0.0510 (-0.0451, 0.1471)	0.0686 (-0.0329, 0.1702)
**HBV Infection**	-0.0510 (-0.0889, -0.0132)*	0.0220 (-0.0052, 0.0492)	0.0190 (-0.0138, 0.0517)
**Vascular Invasion**	-0.0176 (-0.0382, 0.0031)	0.0109 (-0.0015, 0.0233)	0.0077 (-0.0082, 0.0236)
**Child-Pugh Class**	-1.0751 (-6.4846, 4.3344)	0.3390 (-2.8234, 3.5015)	-1.6378 (-4.9507, 1.6751)
**Extrahepatic Metastasis**	-0.0081 (-0.0400, 0.0238)	-0.0021 (-0.0184, 0.0141)	0.0040 (-0.0138, 0.0218)

*P<0.05.

### 3.9 Trial sequential analysis

**Figure 5:**
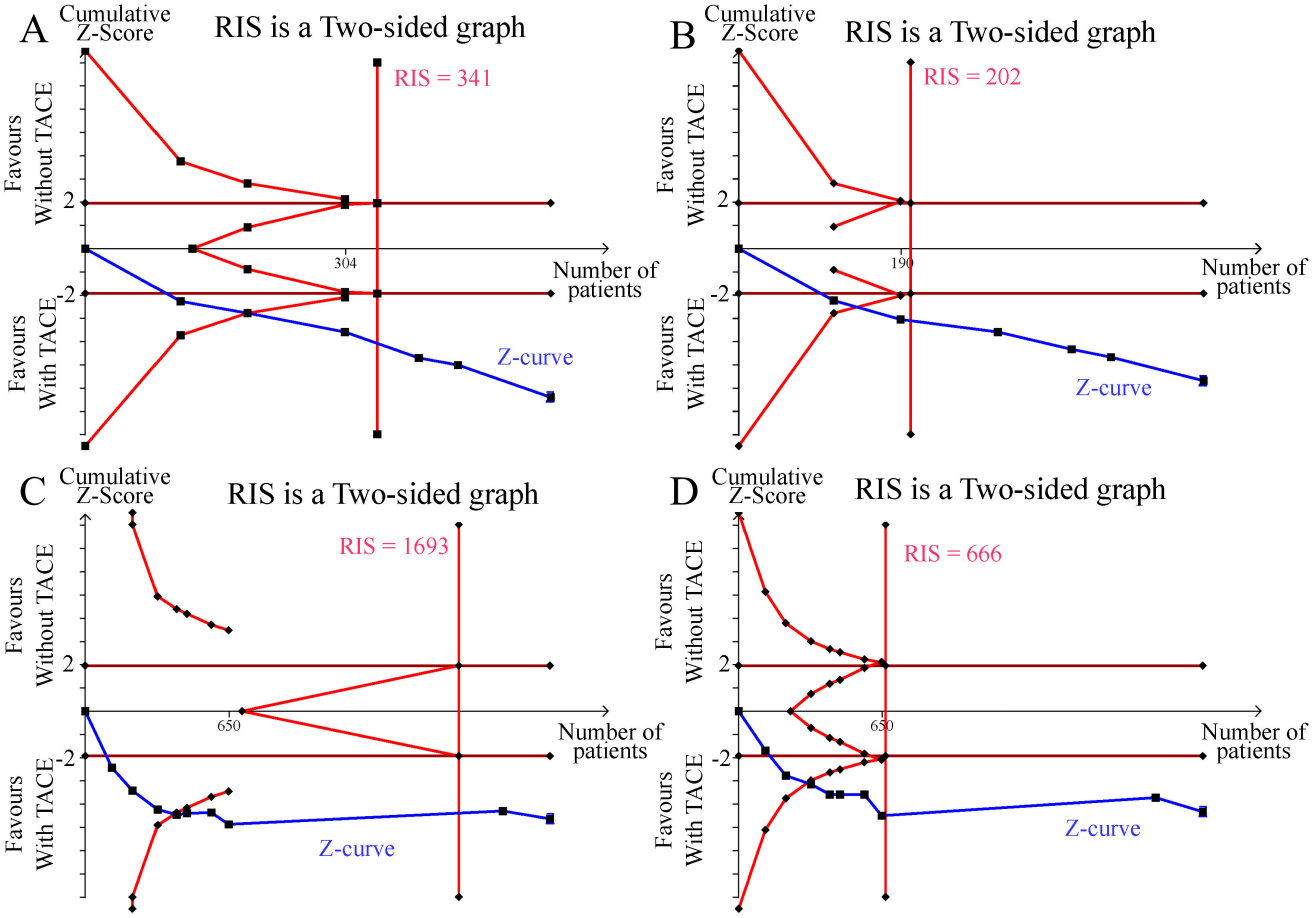
Results of the trial sequential analysis. **(A)** ORR; **(B)** DCR; **(C)** 1-year OS; **(D)** 1-year PFS.

We conducted a TSA for the outcomes of ORR, DCR, 1-OS, and 1-PFS. As illustrated in the figures, the cumulative Z curves in the TSA exceeded both the trial sequential monitoring benefit boundary and the estimated required information size (RIS). This indicates that the meta-analysis has sufficient power to detect statistically significant differences in ORR, DCR, 1-OS, and 1-PFS, and it may not be necessary to conduct further trials to confirm this benefit (**Figure 5**).

### 3.10 Evidence quality

The quality of evidence for the combined results was assessed using the GRADE approach. The findings indicated that the combined results for primary endpoints were of moderate to high quality. In contrast, the evidence quality for the combined results of the secondary endpoints, mOS and mPFS, was found to be lower. Please refer to **Supplementary Figure S9** for further details.

## 4 Discussion

This meta-analysis investigated the efficacy of monotherapy with immune-targeted therapy compared to the combination of immune-targeted therapy and TACE, providing the highest level of evidence to date in related research. The main findings are summarised as follows: compared to immune-targeted therapy alone, the combination of immune-targeted therapy and TACE significantly improved the ORR and DCR in patients with intermediate to advanced HCC, as well as OS and PFS. Even in patients with advanced HCC who exceed the criteria for TACE but do not have main portal vein invasion, the combination of immune-targeted therapy and TACE still further enhances tumour control and prolongs patient survival. TSA further confirmed that there is substantial evidence supporting the conclusions regarding improvements in ORR, DCR, 1-year OS, and 1-year PFS, with the quality of evidence assessed as moderate to high using the GRADE approach.

TACE can lead to a hypoxic tumour microenvironment, which induces the expression of vascular endothelial growth factor, thereby promoting the formation of an immunosuppressive microenvironment (26). Additionally, TACE can induce immunogenic cell death in cancer cells, activating anti-tumour immunity Additionally, TACE can induce immunogenic cell death in cancer cells, activating anti-tumour immunity (10). Thus, there is a robust theoretical basis for the combination of TACE with immune-targeted therapies. Results from the EMERALD-1 trial demonstrated that, in patients with unresectable intermediate-to-advanced HCC, the combination of TACE with Durvalumab and Bevacizumab significantly extended progression-free survival (PFS) compared to TACE alone (HR, 0.77, 95% CI [0.61, 0.98], p = 0.032) (27). Furthermore, a national retrospective cohort study indicated that, among patients with advanced HCC (with vascular invasion or extrahepatic metastasis) receiving first-line immune-targeted therapy, additional TACE treatment further prolonged OS and PFS (3). This meta-analysis supports the findings of the aforementioned study.

In recent years, the concept of TACE inapplicability has emerged (9, 28), particularly for patients meeting certain criteria, such as tumour burden exceeding the up-to-seven criteria or those with diffuse, multinodular infiltrative patterns, pure nodular types with extra-nodular growth, or poorly differentiated HCC, for whom the survival benefits from TACE are suboptimal. For these patients, how to better integrate TACE with systemic therapies requires further exploration, as unnecessary excessive embolisation may lead to deterioration of liver function, adversely affecting prognosis. TACTICS trial provided a theoretical framework for the sequential therapy of Lenvatinib/Sorafenib and TACE, suggesting that the use of anti-VEGF agents prior to initial TACE treatment can correct tumour vasculature and improve drug delivery, thereby enhancing the anti-tumour efficacy of TACE, particularly in patients with tumour burdens exceeding the up-to-seven criteria, showing beneficial effects on PFS and OS (29–31). A proof-of-concept study indicated that patients with HCC deemed unsuitable for TACE may achieve clinical or pathological complete responses through super-selective TACE with curative intent during first-line treatment with atezolizumab/bevacizumab, ultimately attaining a clinical drug-free state (32). In this study, the objective of the TACE procedure was to activate tumour antigen release-mediated tumour-specific immune responses; hence, “incomplete embolisation” was commonly practiced to complement immune-targeted therapies while mitigating liver function impairment (28, 33).

HCC is characterised by a tendency for vascular invasion, often resulting in the formation of portal vein tumour thrombosis (PVTT). At the time of initial diagnosis of HCC, 10% to 40% of patients already exhibit macroscopic PVTT, which significantly impairs their prognosis (34, 35). In the absence of treatment, the median survival time for patients with PVTT is only 2 to 4 months (36). Patients with main portal vein cancer thrombus (mPVTT) represent a subgroup of advanced HCC patients with exceptionally poor prognosis, as portal vein obstruction leads to deteriorating liver function, refractory ascites, and increased risk of gastrointestinal bleeding, thereby elevating mortality risk. It has been reported that, without treatment, the mOS for mPVTT patients is only between 2.2 and 2.7 months (37, 38), which contributes to their frequent exclusion from large phase III clinical trials (6, 39, 40). Evidence supporting survival benefits from TACE predominantly arises from retrospective studies (38, 41, 42). In these studies, cTACE has been shown to extend the median OS of mPVTT patients to approximately 5 months (38, 41), while drug-eluting bead TACE appears to further prolong both OS and TTP (42). The efficacy of combined immunotherapy-targeted therapy and TACE in mPVTT patients remains uncertain. In a real-world study (43), outcomes for patients receiving combined immunotherapy-targeted therapy and TACE did not demonstrate significant differences when compared with those receiving immunotherapy-targeted therapy alone (mOS, 10 months vs. 8 months, p=0.254; mTTP, 4 months vs. 4 months, p=0.404). Multivariate COX regression analysis indicated that treatment allocation was not a key influencing factor for OS (HR, 1.611, 95% CI [0.961, 2.701], p = 0.071). Regrettably, the patient cohort included in the study contained almost no mPVTT patients, thus precluding any definitive conclusions from being drawn.

The strengths of this study include stringent inclusion criteria, incorporating only high-quality PSM studies, which mitigated biases associated with previous retrospective meta-analyses (13). Additionally, we conducted further TSA and evaluated the quality of evidence using the GRADE approach, thereby enhancing the credibility of the combined results. This meta-analysis presents the best available evidence from relevant studies. However, it still has limitations. Firstly, although PSM analysis minimises selection bias, any non-randomised trial is susceptible to inevitable confounding biases that may affect the reliability of the combined results. Secondly, the patient cohorts included in the studies exhibited substantial heterogeneity. Thirdly, four studies only included patients with advanced HCC (BCLC stage C), and 3 studies included patients with recurrent HCC and those who had failed first-line treatment. However, we were unable to perform subgroup analyses, which may limit the generalisability of the combined results in these specific patient subgroups. Fourthly, in two studies, immunotherapy-targeted agents were administered within one week after patients received TACE, while the remaining studies did not provide details on the timing of immunotherapy-targeted agents. The timing of the application of immunotherapy-targeted agents is associated with patient prognosis (32, 44, 45). Therefore, the results of the combined analysis should be interpreted with caution. Lastly, all studies were conducted in China, which restricts the generalisability of the combined findings.

## 5 Conclusion

In the treatment of unresectable HCC with immune-targeted therapy, adding TACE to the treatment regimen can further enhance tumour control and improve patient prognosis. This conclusion is further supported by TSA, which indicates a moderate to high quality of evidence as assessed by the GRADE approach. Even in intermediate or advanced HCC patients who exceed the standard criteria for TACE (excluding those with main portal vein tumour thrombosis), the combination of immune-targeted therapy and TACE can significantly improve patient outcomes compared to immune-targeted therapy alone.

## Data Availability

The original contributions presented in the study are included in the article/**Supplementary Material**. Further inquiries can be directed to the corresponding author.
