# Supplementary material for: The role of TACE in the era of immune-targeted therapy for hepatocellular carcinoma: a meta-analysis based on PSM
Source: Front Immunol. 2025 Apr 2;16:1573834. doi: 10.3389/fimmu.2025.1573834 (PMC12000099; doi:10.3389/fimmu.2025.1573834)
Supplement: Supplementary file 1 [file DataSheet1.docx]

***The role of TACE in the era of immune-targeted therapy for hepatocellular carcinoma: A meta-analysis based on PSM***

Jiahao Li ^a^, Lei Xian ^a^, Xinsen Wang ^a^, Yingnan Liu ^b^, Jiarui Li ^a^

^a^ Department of Interventional Therapy, The First Hospital of Jilin University, Changchun, Jilin Province, People’s Republic of China

^b^ Department of Radiology, The First Hospital of Jilin University, Changchun, Jilin Province, People’s Republic of China

**Corresponding author:**

Jiarui Li

Department of Interventional Therapy, The First Hospital of Jilin University, Changchun, Jilin Province, People’s Republic of China

**E-mail address:** ljr@jlu.edu.cn

**Contents**

[Table. S1. PRISMA Checklist](#_Toc172996540) 3

Table. S2. Literature search criteria 6

[Table. S3. Characteristics of the studies included in the Meta-analysis](#_Toc172996541) 9

[Table. S4. An assessment of the risk of bias was conducted using the ROBINS-I tool](#_Toc172996543) 10

[Figure. S1. Results of the sensitivity analysis](#_Toc172996543) 11

[Figure. S2. Funnel plot adjusted using the trim-and-fill method](#_Toc172996543) 12

[Figure. S3. Subgroup analysis results based on the BCLC staging system.](#_Toc172996543) 13

[Figure. S4. Subgroup analysis results based on the BCLC staging system.](#_Toc172996543) 14

[Figure. S5. Subgroup analysis results based on the proportion of patients with vascular invasion](#_Toc172996543) 15

[Figure. S6. Subgroup analysis results based on the proportion of patients with vascular invasion](#_Toc172996543) 16

[Figure. S7. Subgroup analysis results based on the proportion of patients with extrahepatic metastasis](#_Toc172996543) 17

[Figure. S8. Subgroup analysis results based on the proportion of patients with extrahepatic metastasis](#_Toc172996543) 18

[Figure. S9. The GRADE assessment results for the quality of evidence](#_Toc172996543) 19

Table. S1 PRISMA Checklist

| **Section and Topic** | **Item #** | **Checklist item** | **Location where item is reported** |
| --- | --- | --- | --- |
| **TITLE** | | |  |
| Title | 1 | Identify the report as a systematic review. | 1 |
| **ABSTRACT** | | |  |
| Abstract | 2 | See the PRISMA 2020 for Abstracts checklist. | 2 |
| **INTRODUCTION** | | |  |
| Rationale | 3 | Describe the rationale for the review in the context of existing knowledge. | 2,3 |
| Objectives | 4 | Provide an explicit statement of the objective(s) or question(s) the review addresses. | 2,3 |
| **METHODS** | | |  |
| Eligibility criteria | 5 | Specify the inclusion and exclusion criteria for the review and how studies were grouped for the syntheses. | 4 |
| Information sources | 6 | Specify all databases, registers, websites, organisations, reference lists and other sources searched or consulted to identify studies. Specify the date when each source was last searched or consulted. | 3 |
| Search strategy | 7 | Present the full search strategies for all databases, registers and websites, including any filters and limits used. | 3 |
| Selection process | 8 | Specify the methods used to decide whether a study met the inclusion criteria of the review, including how many reviewers screened each record and each report retrieved, whether they worked independently, and if applicable, details of automation tools used in the process. | Figure1 |
| Data collection process | 9 | Specify the methods used to collect data from reports, including how many reviewers collected data from each report, whether they worked independently, any processes for obtaining or confirming data from study investigators, and if applicable, details of automation tools used in the process. | 5 |
| Data items | 10a | List and define all outcomes for which data were sought. Specify whether all results that were compatible with each outcome domain in each study were sought (e.g. for all measures, time points, analyses), and if not, the methods used to decide which results to collect. | 4,5 |
|  | 10b | List and define all other variables for which data were sought (e.g. participant and intervention characteristics, funding sources). Describe any assumptions made about any missing or unclear information. | 4,5 |
| Study risk of bias assessment | 11 | Specify the methods used to assess risk of bias in the included studies, including details of the tool(s) used, how many reviewers assessed each study and whether they worked independently, and if applicable, details of automation tools used in the process. | 5 |
| Effect measures | 12 | Specify for each outcome the effect measure(s) (e.g. risk ratio, mean difference) used in the synthesis or presentation of results. | 5 |
| Synthesis methods | 13a | Describe the processes used to decide which studies were eligible for each synthesis (e.g. tabulating the study intervention characteristics and comparing against the planned groups for each synthesis (item #5)). | 5 |
|  | 13b | Describe any methods required to prepare the data for presentation or synthesis, such as handling of missing summary statistics, or data conversions. | 5 |
|  | 13c | Describe any methods used to tabulate or visually display results of individual studies and syntheses. | 5 |
|  | 13d | Describe any methods used to synthesize results and provide a rationale for the choice(s). If meta-analysis was performed, describe the model(s), method(s) to identify the presence and extent of statistical heterogeneity, and software package(s) used. | 5 |
|  | 13e | Describe any methods used to explore possible causes of heterogeneity among study results (e.g. subgroup analysis, meta-regression). | 5 |
|  | 13f | Describe any sensitivity analyses conducted to assess robustness of the synthesized results. | 5 |
| Reporting bias assessment | 14 | Describe any methods used to assess risk of bias due to missing results in a synthesis (arising from reporting biases). | 6 |
| Certainty assessment | 15 | Describe any methods used to assess certainty (or confidence) in the body of evidence for an outcome. | 5 |
| **RESULTS** | | |  |
| Study selection | 16a | Describe the results of the search and selection process, from the number of records identified in the search to the number of studies included in the review, ideally using a flow diagram. | 5,6 |
|  | 16b | Cite studies that might appear to meet the inclusion criteria, but which were excluded, and explain why they were excluded. | 5,6 |
| Study characteristics | 17 | Cite each included study and present its characteristics. | Supplementary |
| Risk of bias in studies | 18 | Present assessments of risk of bias for each included study. | Supplementary |
| Results of individual studies | 19 | For all outcomes, present, for each study: (a) summary statistics for each group (where appropriate) and (b) an effect estimate and its precision (e.g. confidence/credible interval), ideally using structured tables or plots. | 6,7 |
| Results of syntheses | 20a | For each synthesis, briefly summarise the characteristics and risk of bias among contributing studies. | 6,7 |
|  | 20b | Present results of all statistical syntheses conducted. If meta-analysis was done, present for each the summary estimate and its precision (e.g. confidence/credible interval) and measures of statistical heterogeneity. If comparing groups, describe the direction of the effect. | 6,7 |
|  | 20c | Present results of all investigations of possible causes of heterogeneity among study results. | 6,7 |
|  | 20d | Present results of all sensitivity analyses conducted to assess the robustness of the synthesized results. | 6,7 |
| Reporting biases | 21 | Present assessments of risk of bias due to missing results (arising from reporting biases) for each synthesis assessed. | 6,7 |
| Certainty of evidence | 22 | Present assessments of certainty (or confidence) in the body of evidence for each outcome assessed. | 9 |
| **DISCUSSION** | | |  |
| Discussion | 23a | Provide a general interpretation of the results in the context of other evidence. | 9 |
|  | 23b | Discuss any limitations of the evidence included in the review. | 10,11 |
|  | 23c | Discuss any limitations of the review processes used. | 11 |
|  | 23d | Discuss implications of the results for practice, policy, and future research. | 11 |
| **OTHER INFORMATION** | | |  |
| Registration and protocol | 24a | Provide registration information for the review, including register name and registration number, or state that the review was not registered. | 3 |
|  | 24b | Indicate where the review protocol can be accessed, or state that a protocol was not prepared. | 3 |
|  | 24c | Describe and explain any amendments to information provided at registration or in the protocol. | 3 |
| Support | 25 | Describe sources of financial or non-financial support for the review, and the role of the funders or sponsors in the review. | 11 |
| Competing interests | 26 | Declare any competing interests of review authors. | 12 |
| Availability of data, code and other materials | 27 | Report which of the following are publicly available and where they can be found: template data collection forms; data extracted from included studies; data used for all analyses; analytic code; any other materials used in the review. | 12 |

# Table. S2. Literature search criteria.

| **Pubmed** |
| --- |
| (((((((((((((((((((((((((("Liver Neoplasms"[Mesh]) OR (Neoplasms, Hepatic[Title/Abstract])) OR (Neoplasms, Liver[Title/Abstract])) OR (Liver Neoplasm[Title/Abstract])) OR (Neoplasm, Liver[Title/Abstract])) OR (Hepatic Neoplasms[Title/Abstract])) OR (Hepatic Neoplasm[Title/Abstract])) OR (Neoplasm, Hepatic[Title/Abstract])) OR (Cancer of Liver[Title/Abstract])) OR (Hepatocellular Cancer[Title/Abstract])) OR (Cancers, Hepatocellular[Title/Abstract])) OR (Hepatocellular Cancers[Title/Abstract])) OR (Hepatic Cancer[Title/Abstract])) OR (Cancer, Hepatic[Title/Abstract])) OR (Cancers, Hepatic[Title/Abstract])) OR (Hepatic Cancers[Title/Abstract])) OR (Liver Cancer[Title/Abstract])) OR (Cancer, Liver[Title/Abstract])) OR (Cancers, Liver[Title/Abstract])) OR (Liver Cancers[Title/Abstract])) OR (Cancer of the Liver[Title/Abstract])) OR (Cancer, Hepatocellular[Title/Abstract])) OR (primary liver cancer[Title/Abstract])) OR (liver primary cancer[Title/Abstract])) AND (((TACE[Title/Abstract]) OR (transarterial chemoembolisation[Title/Abstract])) OR (transarterial chemoembolization[Title/Abstract]))) AND ((((Immunotherapy[Title/Abstract]) OR ("Immunotherapy"[Mesh])) OR ((((((((((((((((((((((((((((((((("Immune Checkpoint Inhibitors"[Mesh]) OR (Checkpoint Inhibitors, Immune[Title/Abstract])) OR (Immune Checkpoint Blockers[Title/Abstract])) OR (Checkpoint Blockers, Immune[Title/Abstract])) OR (Immune Checkpoint Inhibitor[Title/Abstract])) OR (Checkpoint Inhibitor, Immune[Title/Abstract])) OR (CTLA-4 Inhibitors[Title/Abstract])) OR (Cytotoxic T-Lymphocyte-Associated Protein 4 Inhibitors[Title/Abstract])) OR (Cytotoxic T Lymphocyte Associated Protein 4 Inhibitors[Title/Abstract])) OR (Cytotoxic T-Lymphocyte-Associated Protein 4 Inhibitor[Title/Abstract])) OR (Cytotoxic T Lymphocyte Associated Protein 4 Inhibitor[Title/Abstract])) OR (CTLA-4 Inhibitor[Title/Abstract])) OR (CTLA 4 Inhibitor[Title/Abstract])) OR (PD-1 Inhibitors[Title/Abstract])) OR (PD 1 Inhibitors[Title/Abstract])) OR (Programmed Cell Death Protein 1 Inhibitor[Title/Abstract])) OR (Programmed Cell Death Protein 1 Inhibitors[Title/Abstract])) OR (PD-1 Inhibitor[Title/Abstract])) OR (Inhibitor, PD-1[Title/Abstract])) OR (PD 1 Inhibitor[Title/Abstract])) OR (Immune Checkpoint Blockade[Title/Abstract])) OR (Checkpoint Blockade, Immune[Title/Abstract])) OR (Immune Checkpoint Inhibition[Title/Abstract])) OR (Checkpoint Inhibition, Immune[Title/Abstract])) OR (PD-L1 Inhibitors[Title/Abstract])) OR (PD L1 Inhibitors[Title/Abstract])) OR (Programmed Death-Ligand 1 Inhibitors[Title/Abstract])) OR (Programmed Death Ligand 1 Inhibitors[Title/Abstract])) OR (PD-L1 Inhibitor[Title/Abstract])) OR (PD L1 Inhibitor[Title/Abstract])) OR (PD-1-PD-L1 Blockade[Title/Abstract])) OR (Blockade, PD-1-PD-L1[Title/Abstract])) OR (PD 1 PD L1 Blockade[Title/Abstract]))) OR (((((((((((((((Nivolumab[Title/Abstract]) OR (Pembrolizumab[Title/Abstract])) OR (Tislelizumab[Title/Abstract])) OR (Toripalimab[Title/Abstract])) OR (Sintilimab[Title/Abstract])) OR (Camrelizumab[Title/Abstract])) OR (Spartalizumab[Title/Abstract])) OR (Cemiplimab[Title/Abstract])) OR (Atezolizumab[Title/Abstract])) OR (Bevacizumab[Title/Abstract])) OR (Durvalumab[Title/Abstract])) OR (Avelumab[Title/Abstract])) OR (Ipilimumab[Title/Abstract])) OR (Tremelimumab[Title/Abstract])) OR (Cobalimab[Title/Abstract])))) AND ((((((((((("Molecular Targeted Therapy"[Mesh]) OR (Molecular Targeted Therapies[Title/Abstract])) OR (Targeted Therapy, Molecular[Title/Abstract])) OR (Therapy, Molecular Targeted[Title/Abstract])) OR (Targeted Molecular Therapy[Title/Abstract])) OR (Molecular Therapy, Targeted[Title/Abstract])) OR (Targeted Molecular Therapies[Title/Abstract])) OR (Therapy, Targeted Molecular[Title/Abstract])) OR (((((((((((((((((((((((((((((((((((((((((((((((((((((((((((("Angiogenesis Inhibitors"[Mesh]) OR (Angiogenic Inhibitors[Title/Abstract])) OR (Angiostatic Agents[Title/Abstract])) OR (Agents, Angiostatic[Title/Abstract])) OR (Antagonists, Angiogenic[Title/Abstract])) OR (Anti-Angiogenetic Agents[Title/Abstract])) OR (Agents, Anti-Angiogenetic[Title/Abstract])) OR (Anti Angiogenetic Agents[Title/Abstract])) OR (Anti-Angiogenic Drugs[Title/Abstract])) OR (Anti Angiogenic Drugs[Title/Abstract])) OR (Drugs, Anti-Angiogenic[Title/Abstract])) OR (Antiangiogenic Agents[Title/Abstract])) OR (Agents, Antiangiogenic[Title/Abstract])) OR (Inhibitors, Angiogenesis[Title/Abstract])) OR (Inhibitors, Angiogenetic[Title/Abstract])) OR (Inhibitors, Angiogenic[Title/Abstract])) OR (Inhibitors, Neovascularization[Title/Abstract])) OR (Neovascularization Inhibitors[Title/Abstract])) OR (Anti-Angiogenic Drug[Title/Abstract])) OR (Anti Angiogenic Drug[Title/Abstract])) OR (Drug, Anti-Angiogenic[Title/Abstract])) OR (Neovascularization Inhibitor[Title/Abstract])) OR (Inhibitor, Neovascularization[Title/Abstract])) OR (Antiangiogenic Agent[Title/Abstract])) OR (Agent, Antiangiogenic[Title/Abstract])) OR (Angiogenesis Inhibitor[Title/Abstract])) OR (Inhibitor, Angiogenesis[Title/Abstract])) OR (Angiogenetic Antagonist[Title/Abstract])) OR (Antagonist, Angiogenetic[Title/Abstract])) OR (Angiogenetic Antagonists[Title/Abstract])) OR (Antagonists, Angiogenetic[Title/Abstract])) OR (Angiogenetic Inhibitor[Title/Abstract])) OR (Inhibitor, Angiogenetic[Title/Abstract])) OR (Angiogenetic Inhibitors[Title/Abstract])) OR (Angiogenic Antagonists[Title/Abstract])) OR (Angiogenic Antagonist[Title/Abstract])) OR (Antagonist, Angiogenic[Title/Abstract])) OR (Angiogenic Inhibitor[Title/Abstract])) OR (Inhibitor, Angiogenic[Title/Abstract])) OR (Angiostatic Agent[Title/Abstract])) OR (Agent, Angiostatic[Title/Abstract])) OR (Anti-Angiogenetic Agent[Title/Abstract])) OR (Agent, Anti-Angiogenetic[Title/Abstract])) OR (Anti Angiogenetic Agent[Title/Abstract])) OR (Angiogenesis Factor Inhibitors[Title/Abstract])) OR (Factor Inhibitors, Angiogenesis[Title/Abstract])) OR (Inhibitors, Angiogenesis Factor[Title/Abstract])) OR (Angiogenesis Factor Inhibitor[Title/Abstract])) OR (Factor Inhibitor, Angiogenesis[Title/Abstract])) OR (Inhibitor, Angiogenesis Factor[Title/Abstract])) OR (Anti-Angiogenesis Effect[Title/Abstract])) OR (Anti Angiogenesis Effect[Title/Abstract])) OR (Effect, Anti-Angiogenesis[Title/Abstract])) OR (Anti-Angiogenesis Effects[Title/Abstract])) OR (Anti Angiogenesis Effects[Title/Abstract])) OR (Effects, Anti-Angiogenesis[Title/Abstract])) OR (Antiangiogenesis Effect[Title/Abstract])) OR (Effect, Antiangiogenesis[Title/Abstract])) OR (Antiangiogenesis Effects[Title/Abstract])) OR (Effects, Antiangiogenesis[Title/Abstract]))) OR (((((((("Tyrosine Kinase Inhibitors"[Mesh]) OR (Inhibitors, Tyrosine Kinase[Title/Abstract])) OR (Kinase Inhibitors, Tyrosine[Title/Abstract])) OR (Tyrosine Protein Kinase Inhibitors[Title/Abstract])) OR (Tyrosine Kinase Inhibitor[Title/Abstract])) OR (TKI Tyrosine Kinase Inhibitors[Title/Abstract])) OR (TKI[Title/Abstract])) OR (TKIs[Title/Abstract]))) OR ((((((((((((((((((Sorafenib[Title/Abstract]) OR (Galunisertib[Title/Abstract])) OR (Amcasertib[Title/Abstract])) OR (Tepotinib[Title/Abstract])) OR (Lenvatinib[Title/Abstract])) OR (Regorafenib[Title/Abstract])) OR (Tivantinib[Title/Abstract])) OR (Orantinib[Title/Abstract])) OR (Dovitinib[Title/Abstract])) OR (Axitinib[Title/Abstract])) OR (Sunitinib[Title/Abstract])) OR (Linifanib[Title/Abstract])) OR (Nintedanib[Title/Abstract])) OR (Erlotinib[Title/Abstract])) OR (Brivanib[Title/Abstract])) OR (Vandetanib[Title/Abstract])) OR (Cabozantinib[Title/Abstract])) OR (Bevacizumab[Title/Abstract]))) |

| **Study** | **Methods** | **Data source** | **Included patients** | **Control Arm** | **Experimental Arm** | **Size** | **Male (%)** | **Child-pugh A (%)** | **BCLC C (%)** | **HBV+ (%)** | **Vascular invasion (%)** | **Extrahepatic spread (%)** |
| --- | --- | --- | --- | --- | --- | --- | --- | --- | --- | --- | --- | --- |
| C.F-2023 | PSM | Multi-center | Patients with advanced HCC | Atezolizumab/Bevacizumab | Atezolizumab/Bevacizumab + cTACE("on-demand") | 122 | 83.61 | 66.39 | 100.00 | 73.77 | 53.28 | 55.74 |
| L.M-2023 | PSM | Single-center | Patients with intermediate to advanced HCC | Lenvatinib/Sintilimab | Lenvatinib/Sintilimab + DEB-TACE(once every four weeks，"scheduled") | 114 | 87.72 | 78.07 | 59.65 | 91.23 | 28.07 | 39.47 |
| L.H-2023 | PSM | Multi-center | Patients with recurrent HCC | targeted therapy/ PD-1 inhibitors (specific medications unspecified) | targeted therapy/PD-1 inhibitors (specific medications unspecified)+ cTACE("on-demand") | 92 | 90.22 | 72.83 | 78.26 | 75.00 | 53.26 | 30.43 |
| W.J-2023 | PSM | Single-center | Patients with advanced HCC or intermediate HCC with extensive lesions involving both lobes of the liver | Lenvatinib/(pembrolizumab/camrelizumab/sintilimab) | Lenvatinib/(pembrolizumab/camrelizumab/sintilimab)+ cTACE/DEB-TACE("on-demand") | 86 | 87.21 | 79.07 | 82.56 | 87.21 | 56.98 | 54.65 |
| Y.X-2023 | PSM | Single-center | Patients with advanced HCC, patients with recurrent HCC, and patients with HCC who have failed first-line treatment | Regorafenib/(camrelizumab/sintilimab) | Regorafenib/(camrelizumab/sintilimab)+ cTACE/DEB-TACE("on-demand") | 46 | 84.78 | 86.96 | 69.57 | 76.09 | 39.13 | 50.00 |
| C.L-2024 | PSM | Multi-center | Patients with advanced HCC, patients with recurrent HCC, and patients with HCC who have failed first-line treatment | Regorafenib/(camrelizumab/sintilimab) | Regorafenib/(camrelizumab/sintilimab)+ cTACE/DEB-TACE("on-demand") | 112 | 80.36 | 82.14 | 81.25 | 65.18 | 45.54 | 40.18 |
| J.M-2024 | PSM | Single-center | Patients with intermediate to advanced HCC | Lenvatinib/(camrelizumab/sintilimab/tislelizumab) | Lenvatinib/(camrelizumab/sintilimab/tislelizumab)+ cTACE("on-demand") | 78 | 84.62 | 76.92 | 53.85 | 79.49 | 51.28 | 0.00 |
| J.Z-2024 | sIPTW | Multi-center | Patients with advanced HCC | (Anti-VEGF antibody/ tyrosine kinase inhibitors)/ Immune Checkpoints Inhibitors | (Anti-VEGF antibody/ tyrosine kinase inhibitors)/Immune Checkpoints Inhibitors + cTACE/DEB-TACE("on-demand") | 1244 | 86.09 | 81.67 | 100.00 | 84.81 | 70.58 | 58.60 |
| Z.J-2024 | sIPTW | Multi-center | Patients with HCC with portal vein invasion not classified as Vp4 | (Lenvatinib/sorafenib)/ (camrelizumab/sintilimab) | (Lenvatinib/sorafenib)/(camrelizumab/sintilimab)+ cTACE("on-demand") | 215 | 89.30 | 100.00 | 100.00 | 95.81 | 100.00 | 26.51 |

# Table. S3. Characteristics of the studies included in the Meta-analysis.

# Table. S4. An assessment of the risk of bias was conducted using the ROBINS-I tool.

| **Study** | **Pre-intervention** | | **During the intervention** | **Post-intervention** | | | | **Overall bias** |
| --- | --- | --- | --- | --- | --- | --- | --- | --- |
|  | **Bias due to confounding** | **Bias in selection of participants into the study** | **Bias in classification of interventions** | **Bias due to deviations from intended interventions** | **Bias due to missing data** | **Bias in measurement of outcomes** | **Bias in selection of the reported result** |  |
| **C.F-2023** | Low | Low | Low | Low | Low | Low | Low | Moderate |
| **L.M-2023** | Low | Low | Low | Low | Moderate | Low | Low | Moderate |
| **L.H-2023** | Low | Low | Low | Low | Moderate | Low | Low | Moderate |
| **W.J-2023** | Low | Low | Low | Low | Moderate | Low | Low | Moderate |
| **Y.X-2023** | Low | Low | Low | Low | Moderate | Low | Low | Moderate |
| **C.L-2024** | Low | Low | Low | Low | Moderate | Low | Low | Moderate |
| **J.M-2024** | Low | Low | Low | Low | Moderate | Low | Low | Moderate |
| **J.Z-2024** | Low | Low | Low | Low | Low | Low | Low | Low |
| **Z.J-2024** | Low | Low | Low | Low | Low | Low | Low | Low |
| **Overall bias** | Low | Low | Low | Low | Moderate | Low | Low | Moderate |


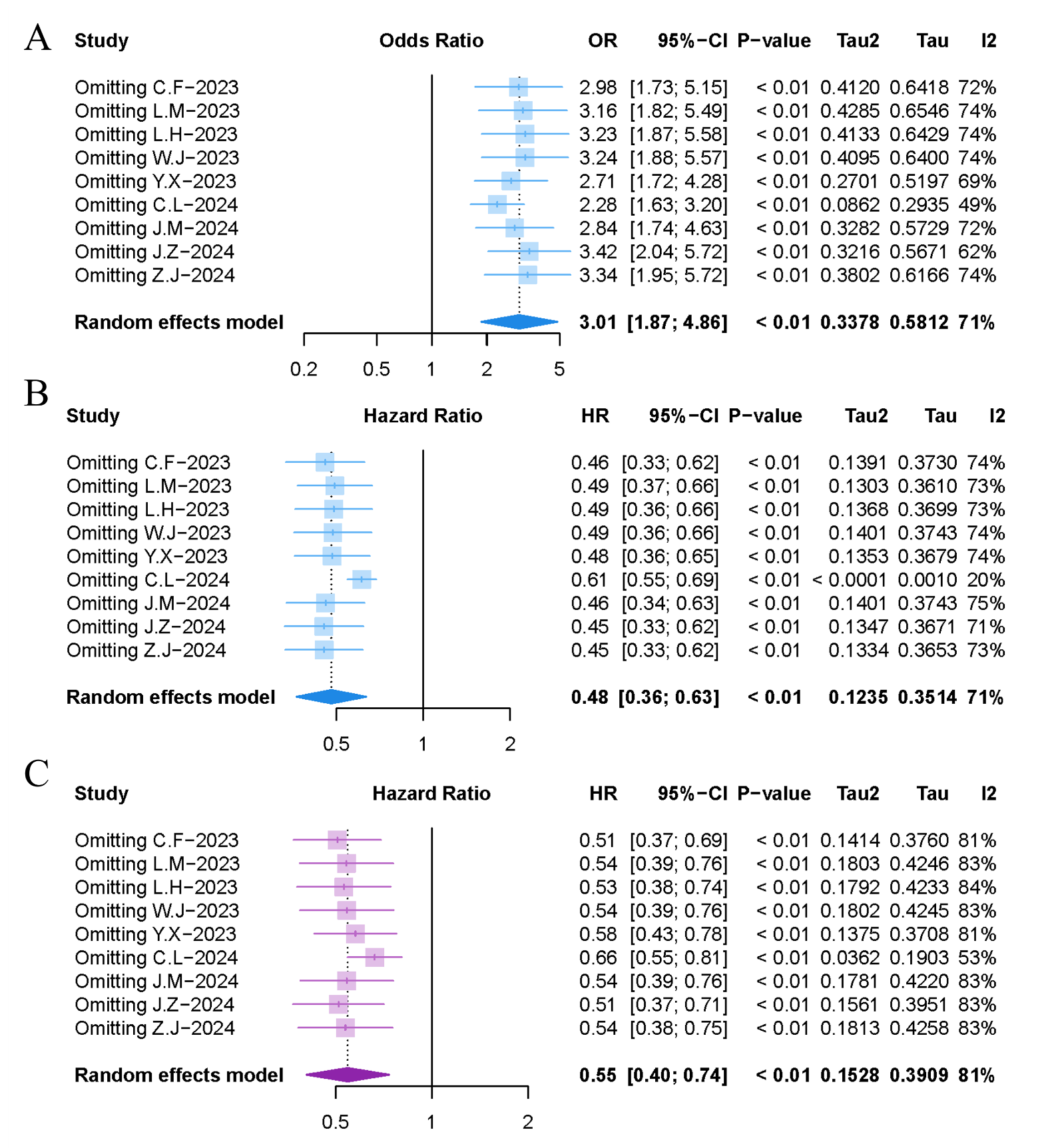


Figure. S1. Results of the sensitivity analysis. (A) 1-year OS; (B) mOS; (C) mPFS.


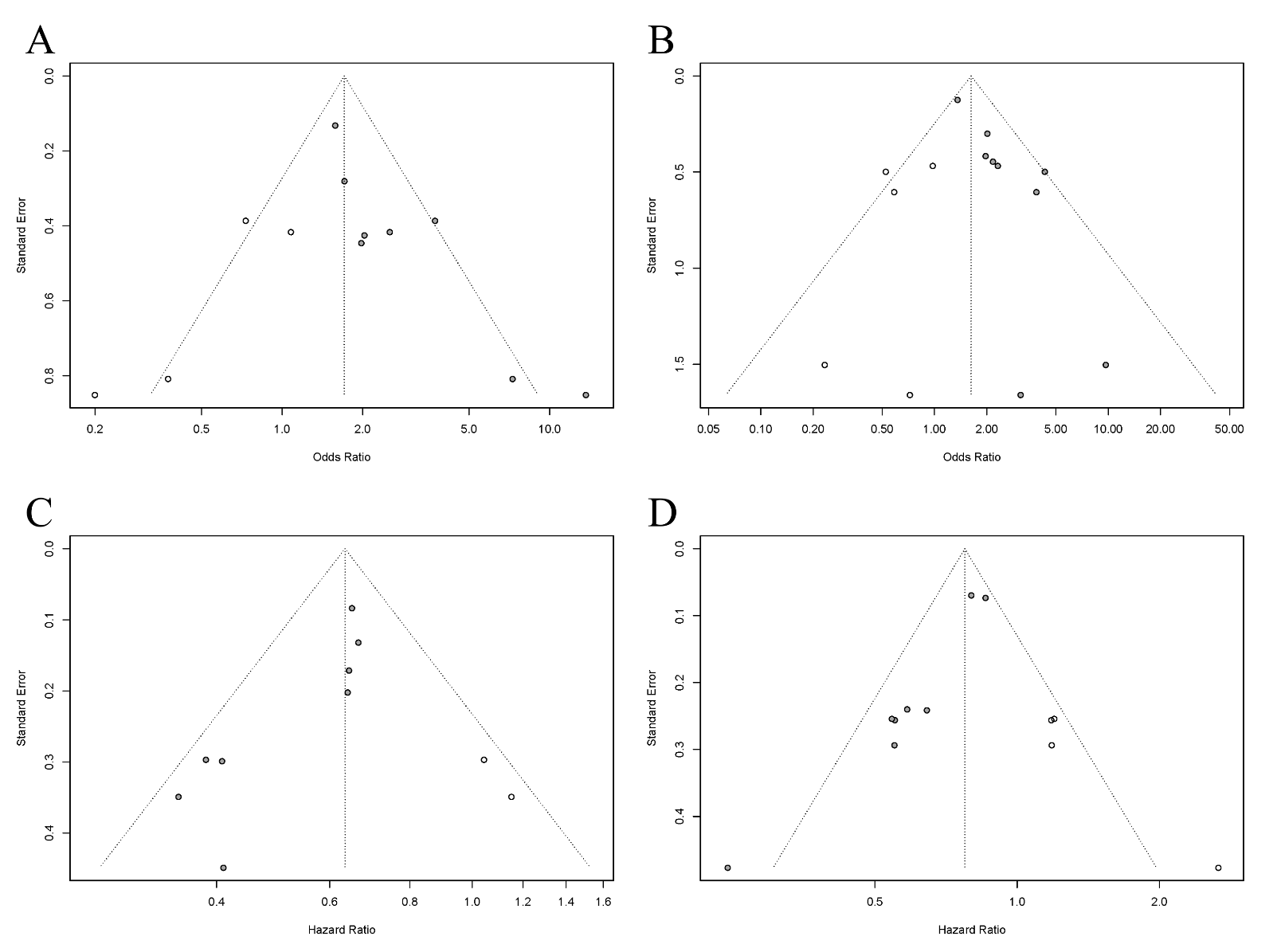


Figure. S2. Funnel plot adjusted using the trim-and-fill method. (A) 1-year OS; (B) 1-year PFS; (C) mOS; (D) mPFS.


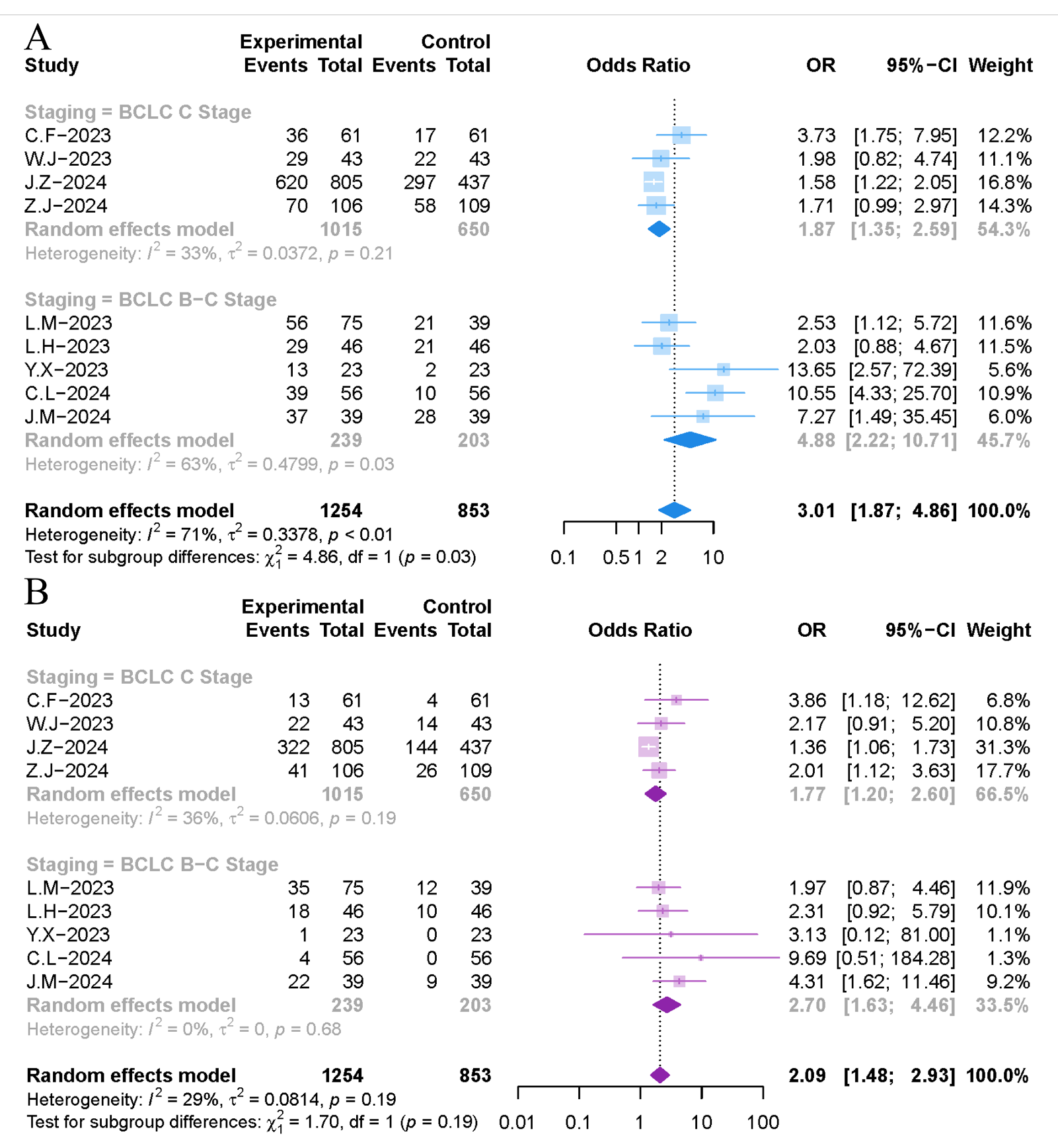


Figure. S3. Subgroup analysis results based on the BCLC staging system. (A) 1-year OS; (B) 1-year PFS.


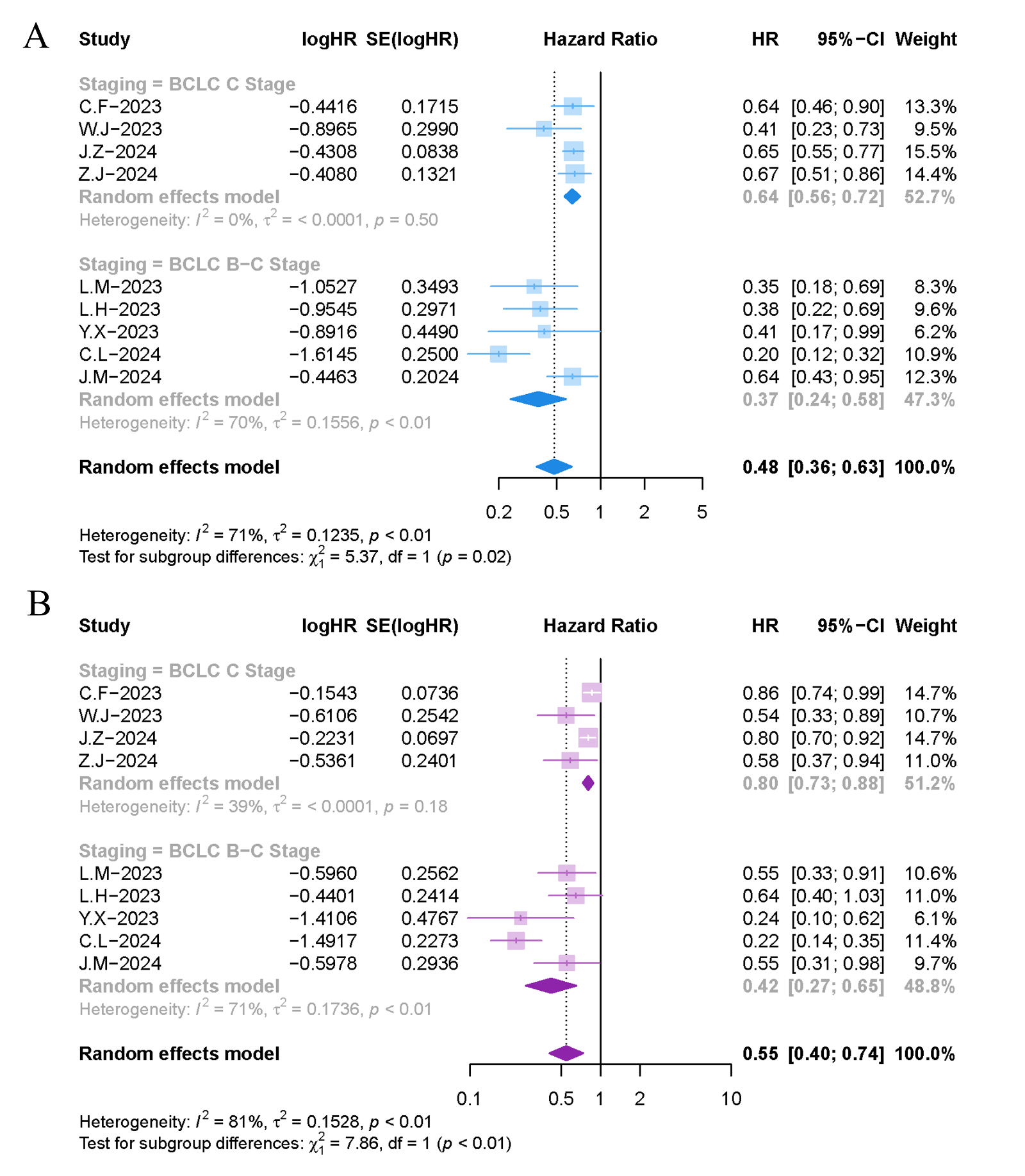


Figure. S4. Subgroup analysis results based on the BCLC staging system. (A) mOS; (B) mPFS.


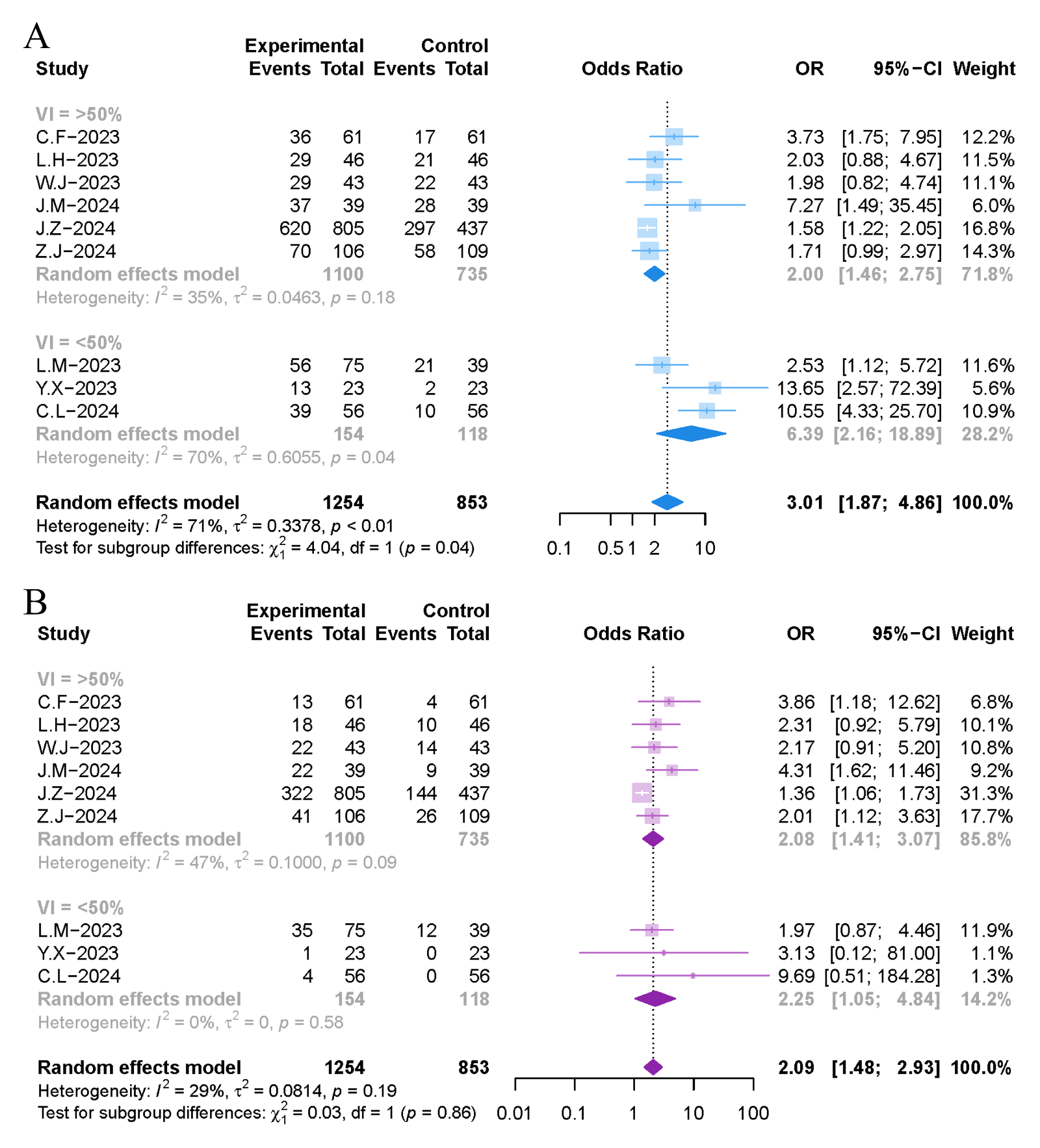


Figure. S5. Subgroup analysis results based on the proportion of patients with vascular invasion. (A) 1-year OS; (B) 1-year PFS.


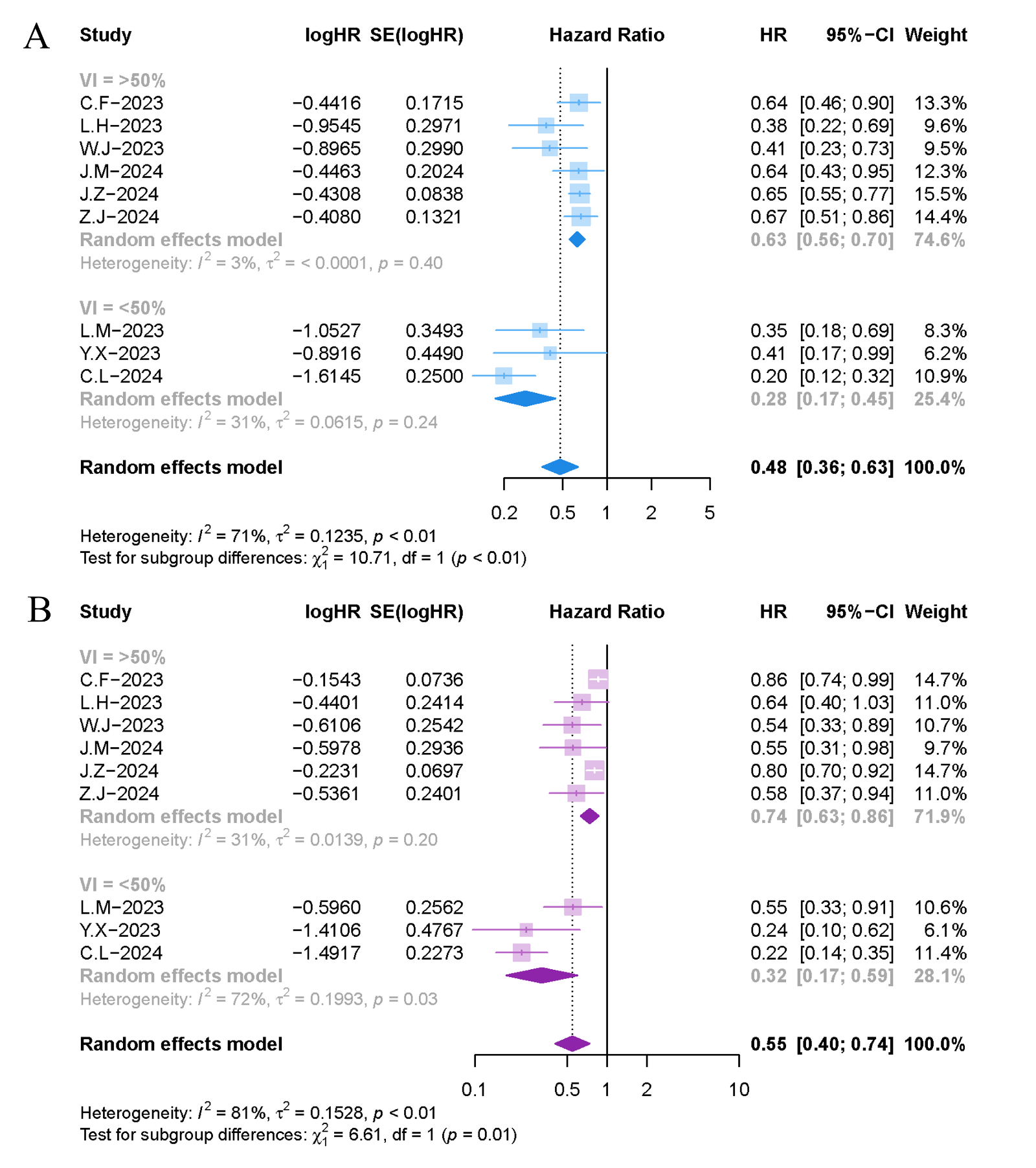


Figure. S6. Subgroup analysis results based on the proportion of patients with vascular invasion. (A) mOS; (B) mPFS.


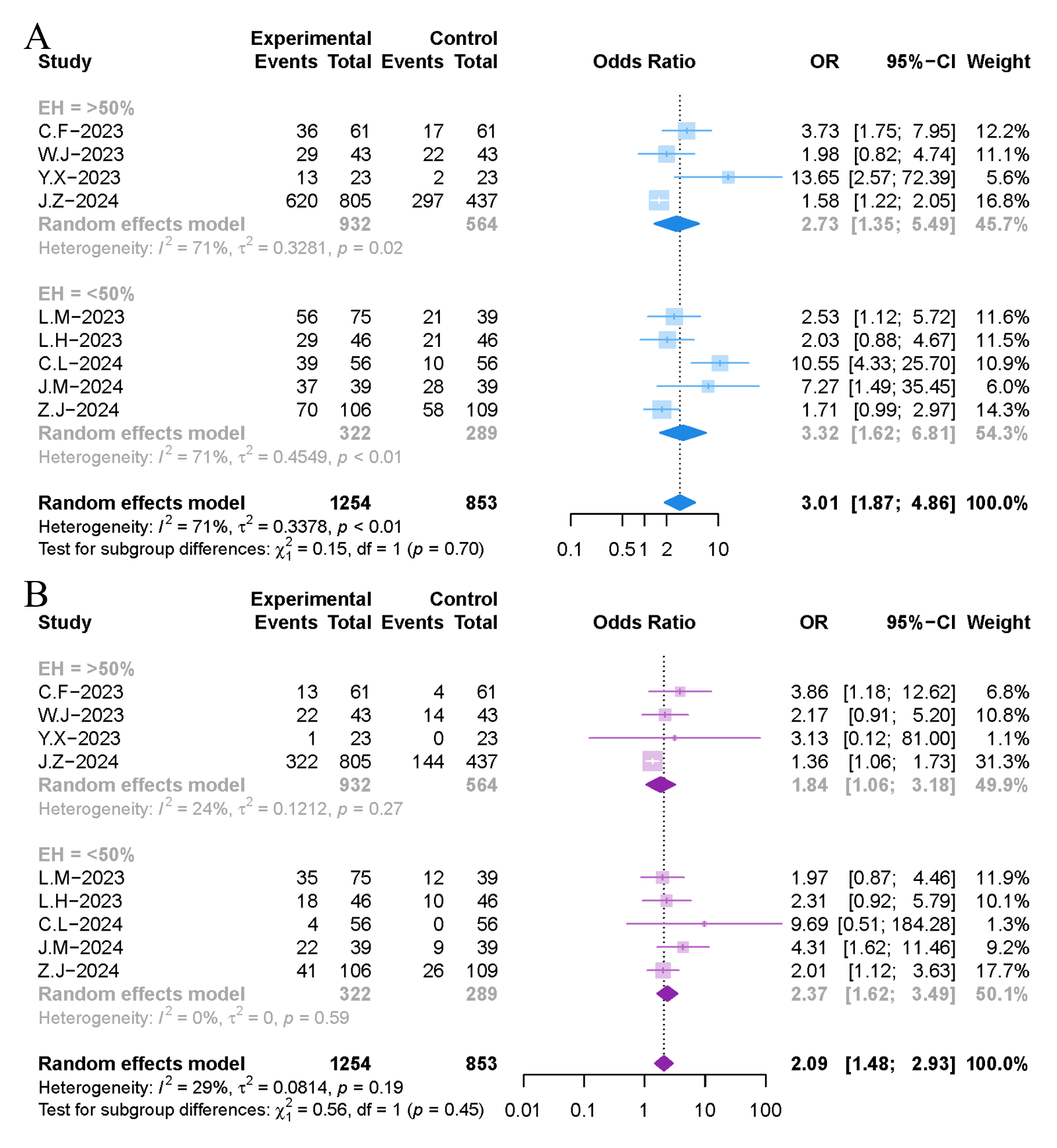


Figure. S7. Subgroup analysis results based on the proportion of patients with extrahepatic metastasis. (A) 1-year OS; (B) 1-year PFS.


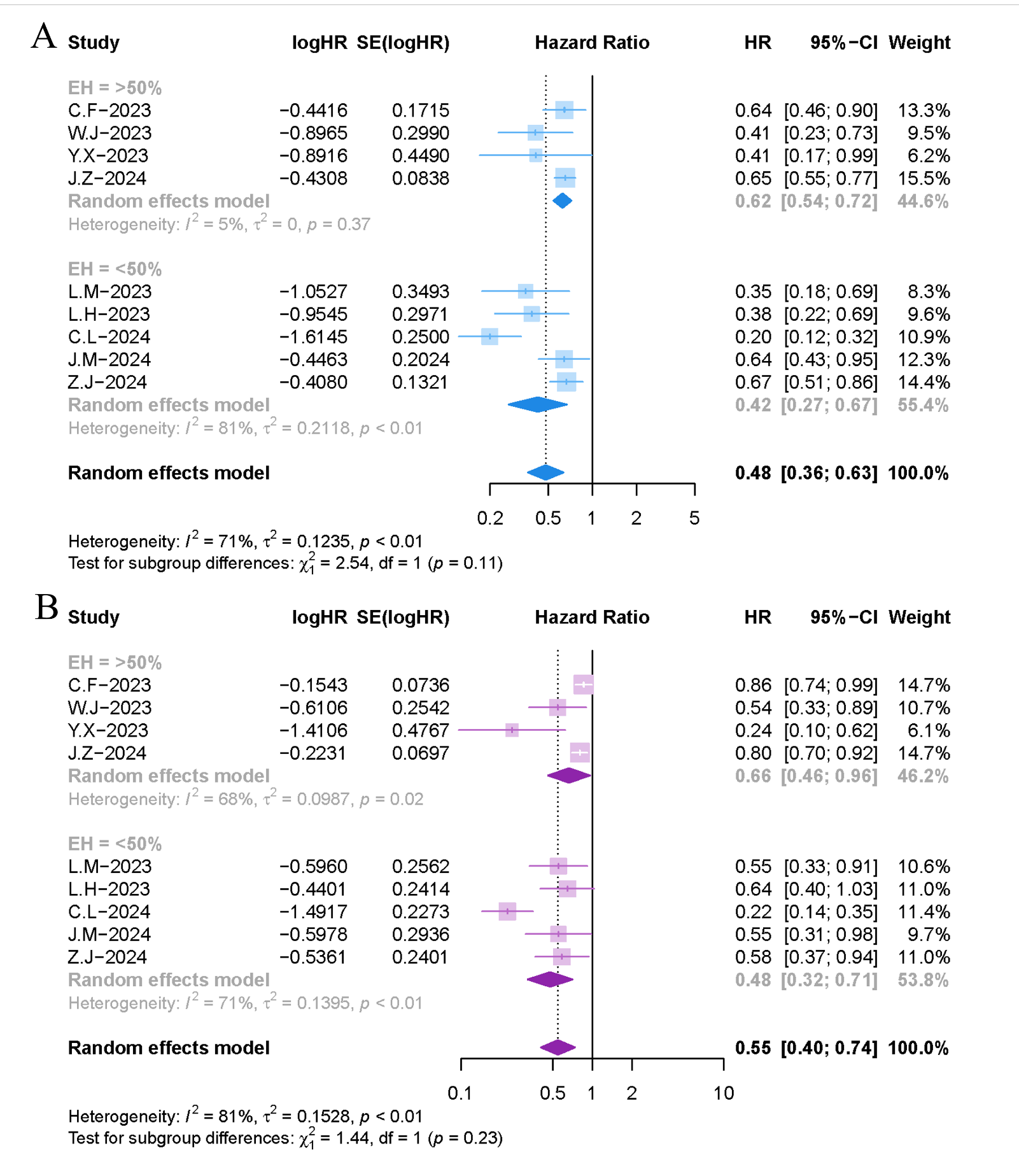


Figure. S8. Subgroup analysis results based on the proportion of patients with extrahepatic metastasis. (A) mOS; (B) mPFS.


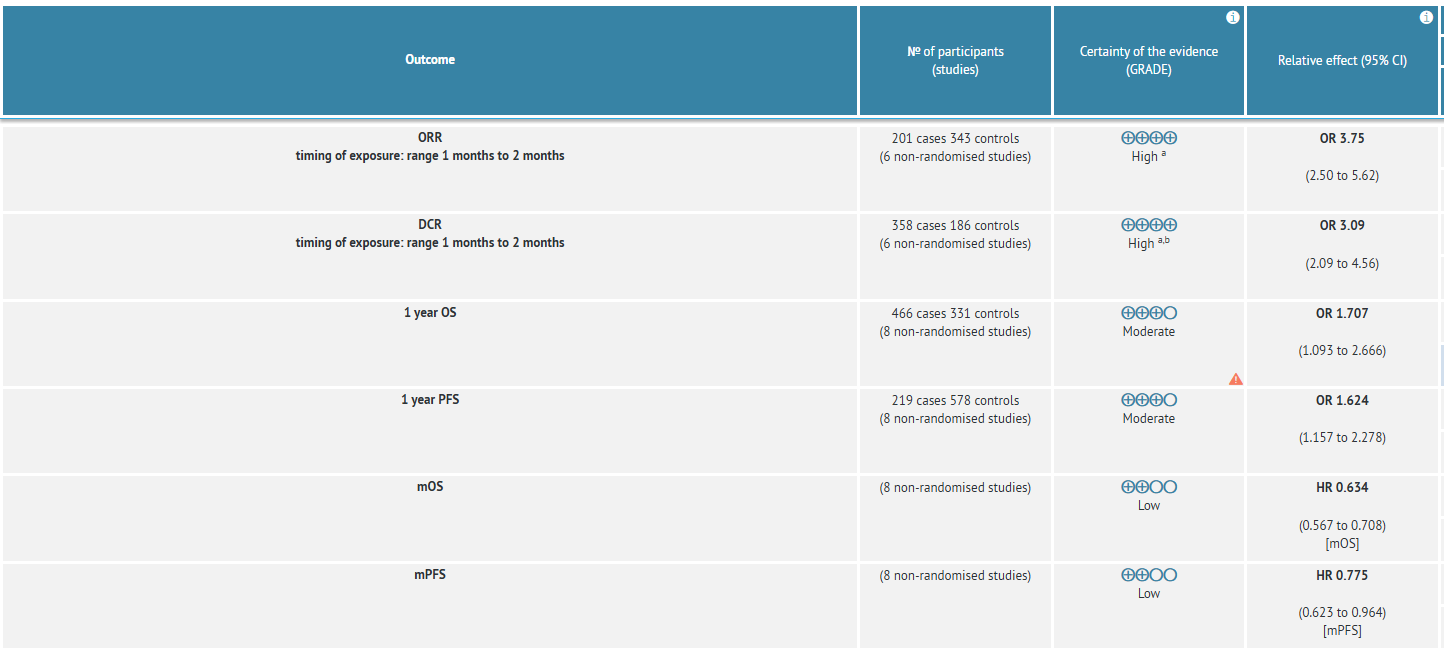


Figure. S9. The GRADE assessment results for the quality of evidence.
